# Combined use of the Consolidated Framework for Implementation Research (CFIR) and the Theoretical Domains Framework (TDF): a systematic review

**DOI:** 10.1186/s13012-016-0534-z

**Published:** 2017-01-05

**Authors:** Sarah A. Birken, Byron J. Powell, Justin Presseau, M. Alexis Kirk, Fabiana Lorencatto, Natalie J. Gould, Christopher M. Shea, Bryan J. Weiner, Jill J. Francis, Yan Yu, Emily Haines, Laura J. Damschroder

**Affiliations:** 1 Department of Health Policy and Management, Gillings School of Global Public Health, The University of North Carolina at Chapel Hill, 1103E McGavran-Greenberg, 135 Dauer Drive, Campus Box 7411, Chapel Hill, NC 27599-7411 USA; 2 Department of Health Policy and Management, Gillings School of Global Public Health, The University of North Carolina at Chapel Hill, 1105C McGavran-Greenberg, 135 Dauer Drive, Campus Box 7411, Chapel Hill, NC 27599-7411 USA; 3 Centre for Practice Changing Research, Ottawa Hospital Research Institute, 501 Smyth Road, Ottawa, Ontario K1H 8L6 Canada; 4 Department of Health Policy and Management, Gillings School of Global Public Health, The University of North Carolina at Chapel Hill, Chapel Hill, NC 27599-7411 USA; 5 End-of-Life, Palliative, and Hospice Care Program, RTI International, 3040 Cornwallis Road, Research Triangle Park, NC 27709 Chapel Hill, USA; 6 School of Health Sciences, City University London, Northampton Square, London, EC1V 0HB UK; 7 Department of Health Policy and Management, University of North Carolina at Chapel Hill, 1102C McGavran-Greenberg Hall, CB# 7411, Chapel Hill, NC 27599-7411 USA; 8 Department of Global Health, Department of Health Services, University of Washington, Box 357965, Seattle, WA 98195-7965 USA; 9 Department of Family Medicine, University of Calgary, 8th Floor, Sheldon M. Chumir Health Centre, 1213-4 Street SW, Calgary, Alberta T2R 0X7 Canada; 10 Department of Health Policy and Management, University of North Carolina at Chapel Hill, 1101B McGavran-Greenberg Hall, CB# 7411, Chapel Hill, NC 27599-7411 USA; 11 VA Ann Arbor Center for Clinical Management Research, Ann Arbor, MI USA; 12 VA Personalizing Options through Veteran Engagement (PROVE) QUERI Program, 2800 Plymouth Road, Building 16, Floor 3, Ann Arbor, MI 48109 USA

**Keywords:** Consolidated Framework for Implementation Research, Theoretical Domains Framework, Implementation theories, Implementation frameworks, Systematic review

## Abstract

**Background:**

Over 60 implementation frameworks exist. Using multiple frameworks may help researchers to address multiple study purposes, levels, and degrees of theoretical heritage and operationalizability; however, using multiple frameworks may result in unnecessary complexity and redundancy if doing so does not address study needs. The Consolidated Framework for Implementation Research (CFIR) and the Theoretical Domains Framework (TDF) are both well-operationalized, multi-level implementation determinant frameworks derived from theory. As such, the rationale for using the frameworks in combination (i.e., CFIR + TDF) is unclear. The objective of this systematic review was to elucidate the rationale for using CFIR + TDF by (1) describing studies that have used CFIR + TDF, (2) how they used CFIR + TDF, and (2) their stated rationale for using CFIR + TDF.

**Methods:**

We undertook a systematic review to identify studies that mentioned both the CFIR and the TDF, were written in English, were peer-reviewed, and reported either a protocol or results of an empirical study in MEDLINE/PubMed, PsycInfo, Web of Science, or Google Scholar. We then abstracted data into a matrix and analyzed it qualitatively, identifying salient themes.

**Findings:**

We identified five protocols and seven completed studies that used CFIR + TDF. CFIR + TDF was applied to studies in several countries, to a range of healthcare interventions, and at multiple intervention phases; used many designs, methods, and units of analysis; and assessed a variety of outcomes. Three studies indicated that using CFIR + TDF addressed multiple study purposes. Six studies indicated that using CFIR + TDF addressed multiple conceptual levels. Four studies did not explicitly state their rationale for using CFIR + TDF.

**Conclusions:**

Differences in the purposes that authors of the CFIR (e.g., comprehensive set of implementation determinants) and the TDF (e.g., intervention development) propose help to justify the use of CFIR + TDF. Given that the CFIR and the TDF are both multi-level frameworks, the rationale that using CFIR + TDF is needed to address multiple conceptual levels may reflect potentially misleading conventional wisdom. On the other hand, using CFIR + TDF may more fully define the multi-level nature of implementation. To avoid concerns about unnecessary complexity and redundancy, scholars who use CFIR + TDF and combinations of other frameworks should specify how the frameworks contribute to their study.

**Trial registration:**

PROSPERO CRD42015027615

**Electronic supplementary material:**

The online version of this article (doi:10.1186/s13012-016-0534-z) contains supplementary material, which is available to authorized users.

## Background

Scholars seeking to study innovation implementation in healthcare have over 60 conceptual frameworks to guide their work [1]. Frameworks can guide implementation, facilitate the identification of determinants of implementation, guide the selection of implementation strategies, and inform all phases of research by helping to frame study questions and hypotheses, anchor background literature, clarify constructs to be measured, depict relationships to be tested, and contextualize results [2, 3]. Frameworks provide a common language, allowing for cumulative evidence to develop.

Implementation frameworks may differ from one another in a number of ways. First, they may serve different *purposes*: to describe/guide the implementation process as a whole (e.g., the Knowledge to Action framework [4]), to identify determinants of implementation (e.g., the Consolidated Framework for Implementation Research (CFIR [5]), the Theoretical Domains Framework (TDF [6])), or to evaluate implementation (e.g., Reach Effectiveness Adoption Implementation Maintenance [7]). Second, implementation frameworks differ in the *conceptual level* at which they focus, with some focused on a single level (e.g., organizational, team, individual) and others being multi-level [1, 8]. Third, they differ in their *degree of theoretical heritage*, ranging from emergent, context-specific conceptual frameworks to theoretical frameworks that describe and/or combine explanations derived from multiple evidence-based theories (e.g., the exploration, adoption decision/preparation, active implementation, sustainment framework). Fourth, they may differ in their degree of *operationalizability*, with some including definitions, tools, and suggested methodological approaches to facilitate use and promote consistent application [1]. For example, the CFIR has an online technical assistance website (www.cfirguide.org) with sample interview questions that tap included specific constructs, and Michie et al. (2005), which introduces the TDF, contains sample interview questions for each TDF domain as well as a recently developed quantitative questionnaire [6, 9]. Atkins et al. have a manual for TDF application currently under review for publication (personal communication, Lou Atkins, November 15, 2016).

A key challenge for researchers and practitioners is how to select from among the growing number of frameworks [10]. In many cases, a single framework can be used to address study needs. In some cases, scholars may use multiple frameworks because a single framework cannot comprehensively address study needs. Scholars may need to use multiple frameworks to address multiple study *purposes* (e.g., to identify determinants and inform evaluation) or *conceptual levels* (i.e., multi-level studies), to account for multiple *theoretical perspectives*, or to adequately *operationalize* key concepts. In contrast, if a single framework is sufficient for addressing study needs, using multiple frameworks may threaten the scientific principle of parsimony, potentially resulting in unnecessary complexity and redundancy, particularly if each included framework does not contribute some unique content (e.g., purpose, conceptual level, theoretical perspective, operationalization).

To avoid concerns that using multiple frameworks introduces unnecessary complexity and redundancy, scholars should provide a clear rationale for using multiple frameworks. Analyzing studies that use both the CFIR and the TDF (hereafter, *CFIR + TDF*) may be instructive for understanding scholars’ rationales for using multiple frameworks because of these frameworks’ apparent similarities: The CFIR and the TDF are both well-operationalized, multi-level implementation determinant frameworks derived from theory. The CFIR includes 39 constructs (i.e., discrete theoretical concepts) arranged across five domains (i.e., groups of conceptually related constructs), emphasizing determinants of implementation that may be active primarily, though not exclusively, at the collective (e.g., organization) level. Domains include intervention characteristics (e.g., adaptability), outer setting (e.g., patient needs and resources), inner setting (e.g., culture), and process (e.g., planning). One domain, characteristics of individuals, focuses on individual-level constructs (e.g., self-efficacy). The CFIR has been applied to a diverse array of studies that have investigated mental health workers’ views of a health self-management program, identified determinants of successful implementation of evidence-based practices in public health agencies, designed a tailored intervention strategy to improve hospital services for children, and evaluated success of an implementation trial to improve uptake of a re-engagement program for patients with mental illness in Veterans Affairs medical centers, among others [11].

The TDF is another commonly used implementation determinant framework that includes 128 constructs in 12 domains derived from 33 theories of behavior change [6]. The TDF provides a high level of elaboration for constructs related to individual level change though it also includes collective (e.g., organization) level constructs [12]. TDF domains include knowledge (e.g., of scientific rationale for implementation); skills (e.g., ability); social/professional role and identity (e.g., group norms); beliefs about capabilities (e.g., self-efficacy); beliefs about consequences (e.g., outcome expectancies); motivation and goals (e.g., intention); memory, attention, and decision processes (e.g., attention control); environmental context and resources (e.g., resources); social influences (e.g., leadership); emotion (e.g., burnout); behavioral regulation (e.g., feedback); and nature of the behavior (e.g., routine). The TDF has also been applied in numerous studies, including process evaluation of a Canadian CT head rule trial, a qualitative study of factors influencing mild traumatic brain injury in the emergency department, barriers and facilitators of interventions to engage pregnant women in smoking cessation, and investigation of perceptions about pre-operative testing in low-risk patients [13–16].

We are aware of (and, in the case of BP, FL, NG, and JF, have authored) studies that have used CFIR + TDF; however, given the apparent similarities between the CFIR and the TDF in terms of purpose, level, degree of theoretical heritage, and operationalizability, the rationale for using CFIR + TDF is not readily apparent. The objective of this study is to elucidate the rationale for using CFIR + TDF. To achieve this objective, we describe (1) published studies that have used CFIR + TDF, (2) how they used CFIR + TDF (e.g., to address multiple study purposes or conceptual levels), and (3) their stated rationale for using CFIR + TDF. In fulfilling this objective, we aim to inform the judicious use of CFIR + TDF and combinations of other frameworks in future implementation studies. When necessary, using multiple frameworks to address study needs may help to limit the proliferation of frameworks and the related fragmentation of knowledge by ensuring that existing frameworks continue to be used, evaluated, and refined and by avoiding segmentation of the field through the use of a single preferred framework over another [1, 17, 18]. Using multiple frameworks may curtail “pseudoinnovation,” wherein perceived advances in framework development are more aptly characterized as reinvention rather than true innovation [18]. In addition, studies that use multiple frameworks may yield more practically relevant results, particularly if the frameworks selected can help to conceptualize implementation at multiple levels [8, 19]. Perhaps equally important, the use of multiple frameworks may represent an opportunity to move implementation science toward greater interdisciplinarity.

## Methods

Our systematic review was conducted in accordance with the Preferred Reporting Items for Systematic Reviews and Meta-Analyses (PRISMA) statement and checklist (Additional file 1), using the accompanying explanation and elaboration document. The review protocol was registered with the International Prospective Register of Systematic Reviews (PROSPERO) on November 3, 2015 and updated on December 12, 2016 (registration number CRD42015027615).

### Search strategy

To identify studies that used CFIR + TDF, we searched for published articles that referred to both the “Consolidated Framework for Implementation Research” (or “CFIR”) and the “Theoretical Domains Framework” (or “TDF”) in the full text in the following databases: MEDLINE/PubMed, PsycInfo, Web of Science, and Google Scholar. The TDF was published in the 2005 article “Making psychological theory useful for implementing evidence based practice: a consensus approach” (Michie et al. 2005 [6]), but it was not named as the Theoretical Domains Framework until 2012 [12]. To capture records that used the CFIR and referenced Michie et al. (2005) [6], possibly representing the use of both the CFIR and the TDF before it was named as such, we also searched PsycInfo, Web of Science, and Google Scholar for records that referred to both the “Consolidated Framework for Implementation Research” (or “CFIR”) and “Making psychological theory useful for implementing evidence based practice: a consensus approach [6].” (We did not search PubMed because it does not search references.) We conducted these searches first in December 2015 and again in October 2016.

### Inclusion criteria

To be included in the study, records were required to mention both the CFIR and the TDF, be written in the English language and peer-reviewed, and report either a protocol for or results of an empirical study.

### Study selection process

SB, AK, and YY selected records for inclusion in the study. These authors conducted title, abstract, and full-text review, searching for evidence of CFIR + TDF use. Discrepancies were resolved through discussions between the three authors and, when necessary, BP until consensus was reached. During this process, 65 records were excluded because they did not report empirical research, were not published in English, or did not use both frameworks. SB and either AK or YY then reviewed full text of the remaining 12 records, confirming evidence of CFIR + TDF use in each record.

### Data extraction and analysis

Given our a priori interest in understanding why and how CFIR + TDF has been used, we used a framework analysis approach [20]. In general, the framework analysis approach allows researchers to analyze qualitative data in a matrix format (i.e., Excel workbook) consisting of rows (cases), columns (codes), and cells (summarized data [21]). We adopted a framework analysis approach that included five key phases: familiarization, identifying a thematic framework, indexing, charting, and mapping and interpretation. First, in the *familiarization* phase, we reviewed included studies and familiarized ourselves with the literature base. Second, we *identified a thematic framework* based on our specific research objectives. This thematic framework served as the columns (codes) for data abstraction. To describe studies to which researchers have applied CFIR + TDF, our thematic framework included study objective, design, setting, unit of analysis, and outcomes assessed. Consistent with our study objectives, our thematic framework also included authors’ stated rationale for using CFIR + TDF and how CFIR + TDF was used (i.e., explicit rationale for using CFIR + TDF, specifically, related to one or more of the dimensions listed in Table 2 or another dimension that authors identified as a rationale for using CFIR + TDF). Next, in the *indexing and charting* phases, we abstracted text selections from included articles and placed them into the appropriate cells within our framework. Indexing and charting of all included articles was completed by two authors (SB, AK). All discrepancies in the indexing and charting phase were discussed until consensus was reached. Finally, in the *mapping and interpretation* phase, summarized data from each cell were analyzed to address each research question ((1) what studies have used CFIR + TDF, (2) how they used CFIR + TDF (e.g., framing, data collection, analysis), and (3) their stated rationale for using CFIR + TDF). Themes related to each research question were discussed among SB, BP, and AK until consensus was reached.

## Results

**Fig. 1:**
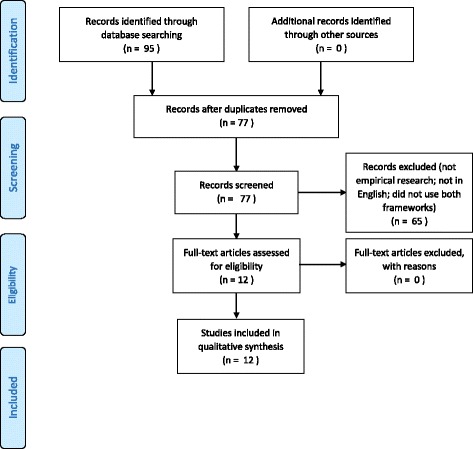
PRISMA flow diagram

Our search yielded 95 publications. We removed 18 duplicates, leaving 77 for screening; of these, we excluded 65 publications because they did not mention both the CFIR and the TDF, were not written in English, or did not report a protocol or results of empirical studies (see Fig. 1). We identified 12 CFIR + TDF articles; the final list of included studies comprised five protocols for empirical studies (Gould [22], Prior [23], Manca [24], Graham-Rowe [25], Sales [26]) and seven completed empirical studies (Murphy [27], English [28], Bunger [29], Moullin [30], Newlands [31], Templeton [32], Elouafkaoui [33]).

### Description of studies that have used CFIR + TDF

Table 1 displays characteristics of included studies: objective, setting, intervention phase (i.e., design, feasibility/piloting, implementation, and evaluation), design, methods, data sources, unit of analysis, and outcomes assessed. Throughout the description of studies that have used CFIR + TDF that follows, we incorporate descriptions of how the studies used CFIR + TDF.

**Table 1:** Study characteristics

Study	Objective	Setting	Phase of intervention	Study design	Methods	Data collection	Data analysis	Unit of analysis	Outcomes assessed
Bunger et al. [29]	To investigate how a learning collaborative focusing on trauma-focused cognitive behavioral therapy impacted advice seeking patterns between clinicians and key learning sources	Behavioral Health Agencies (USA)	Evaluation	Observational	Quantitative	Questionnaires	Social network analysis	Individual and organization	Change in professional networks
Elouafkaoui et al. [33]	To analyze the impact of individualized audit and feedback interventions on dentists’ antibiotic prescribing rates	NHS general dental practices in Scotland	Implementation and evaluation	Experimental	Cluster randomized controlled trial; comparative effectiveness and process evaluation	Prescribing and claims data	Single principle analysis, analyses of covariance, intra-cluster correlations	Organization	Total number of antibiotic items dispensed per 100 NHS treatment claims over 12 months after intervention
English [28]	To design an intervention to improve district hospital services for children	Hospitals (Kenya)	Design	Observational	N/A	Environmental scans/literature searches; a priori knowledge about context	Repeatedly moving backwards and forwards between identified causes, proposed interventions, identified theory, and knowledge of the existing context to develop the intervention	N/A	N/A
Gould et al. [22]^a^	Design 2: theoretically enhanced audit and feedback interventions and investigate their feasibility and acceptability	Hospitals (England)	Feasibility assessment, piloting	Observational	Mixed	Study A: existing feedback documents (e.g., written reports, action planning templates) Study B: semi-structured interviews and observations Study C: semi-structured interviews, observations, surveys	Study A: structured content analysis Study B: qualitative case study analysis Study C: content analysis of interviews and descriptive statistics from questionnaires	Organization	Specific beliefs relating to ordering blood transfusion, determinants of implementation
Graham-Rowe et al. [25]^a^	To identify and synthesize modifiable barriers and enablers in screening for diabetic retinopathy	Multiple	Evaluation	Systematic review	Systematic literature search	Qualitative and quantitative data extracted from identified literature	Theory-based structured content analysis	Individual and organization	The potential role and relative importance of each TDF and CFIR domain in influencing retinopathy screening attendance; plus variations in barriers and enablers across demographic groups
Manca et al. [24]^a^	To implement and evaluate the Building on Existing Tools to Improve Chronic Disease Prevention and Screening in Primary Care program	Primary care (Canada)	Evaluation	Observational	Mixed	Descriptive data; semi-structured interviews	Descriptive statistics; qualitative content analysis	Individual	Program reach, effectiveness, adoption, maintenance
Moullin et al. [30]	To investigate professional service implementation in community pharmacy, to contextualize and advance a generic implementation framework	Community pharmacies (Australia)	Evaluation	Observational	Qualitative	Semi-structured one-on-one interviews	Framework analysis	Individual and organization	General themes surrounding the process of implementation, and influences on implementation
Murphy et al. [27]	Design and implement a capacity-building program to enhance pharmacist’ roles in mental health care	Pharmacies (Canada)	Design	Observational	N/A	Environmental scans/literature searches; a priori knowledge about context	Identified target behavior, conducted a capability-opportunity-motivation and behavior assessment, and identified specific behavior change techniques	N/A	N/A
Newlands et al. [31]	To elucidate barriers and facilitators of using local measures instead of prescribing antibiotics to manage dental infections	NHS general dental practices in (Scotland)	Evaluation	Observational	Qualitative	Semi-structured one-on-one interviews	Theory-based structured content analysis	Individual	Self-reported barriers and facilitators of using just local measures, and not antibiotics, to treat dental infections
Prior et al. [23]^a^	Compare effectiveness of and evaluate processes associated with individualized audit and feedback strategies for translating evidence-based guidelines on antibiotic prescribing into dentistry practice	General dentist practices (Scotland)	Implementation	Experimental	Partial factorial cluster randomized controlled trial; comparative effectiveness, process evaluation	Claims data, semi-structured interviews	Analysis of covariance and content analysis	Organization	Number of antibiotic items dispensed, specific beliefs regarding prescribing behavior, barriers and facilitators to implementation
Sales et al. [26]^a^	Determine the context, barriers, and facilitators to providing advanced care planning and goals of care conversations with veterans, to support providers in meeting a new system-wide mandate for these conversations	Veterans Affairs nursing homes, Veterans Affairs home-based primary care programs in five regional Veterans Affairs networks (USA)	Design and implementation	Observational	Mixed	Context and barrier and facilitator assessments	Interrupted time series/segmented regression analysis with matched comparisons	Individual and organization	(1) Proportion of veterans who have documented goals of care conversations after admission; (2) variation in goals of care conversation practice measures; (3) development of tools to improve implementing goals of care conversations
Templeton et al. [32]	Identify patient-, organization-, and system-level factors influencing dental caries management	NHS primary care dentist offices in (Scotland)	Evaluation	Observational	Mixed	Questionnaires assessing current practices and beliefs sent to 651 dentists; eight in-depth case studies that observed routine dental visits and interviewed providers and patients	Descriptive statistics, univariate analyses, logistic regressions, and qualitative content analysis	Individual and organization	Perceptions of barriers and facilitators to improve caries prevention and management, from the point of view of patients, providers, the dental practices themselves and policy-makers

*CFIR* Consolidated Framework for Implementation Research [5], *TDF* Theoretical Domains Framework [6], *NHS* National Health Service

^a^Study protocol

#### Objective

All studies’ objectives were related to interventions to improve health care. Two completed studies and two protocols described intervention design and/or implementation plans (Gould [22], Sales [26], Murphy [27], English [28]); one protocol described a comparison of the effectiveness of an intervention relative to standard care (Prior [23]), and two protocols and five completed studies evaluated interventions (Manca [24], Sales [26], Bunger [29], Moullin [30], Newlands [31], Templeton [32], Elouafkaoui [33]). Several of the included studies had multiple objectives (e.g., effectiveness study and concurrent qualitative process evaluation [23, 33]).

#### Setting

All study settings were healthcare organizations. Two were hospitals (Gould [22], English [28]). Other settings included pharmacy, dentistry, primary care, and behavioral health agencies. Studies were conducted in Australia (1), Canada (2), England (1), Kenya (1), Scotland (4), and the USA (2).

#### Intervention phase

In the included studies, CFIR + TDF was applied across all phases of an intervention (i.e., design, feasibility/piloting, implementation, and evaluation; see Table 1). Two completed studies and two protocols (Gould [22], Sales [26], Murphy [27], English [28]) used the frameworks in the intervention design phase. One of these protocols (Gould [22]) also planned to apply CFIR + TDF in the feasibility/piloting phase to identify current practice of healthcare professionals within the intervention target healthcare setting and identify barriers and facilitators to performing the target behavior. The other protocol (Sales [26]) also planned to apply CFIR + TDF in the implementation phase. Another protocol (Prior [23]) was focused only on the implementation phase, proposing to use CFIR + TDF to assess acceptability of the intervention and identify barriers and facilitators to the intervention target behavior. Ostensibly, protocols may yield future intervention development papers and results papers that apply CFIR + TDF to additional intervention phases. Five completed studies and two protocols applied CFIR + TDF to the intervention evaluation phase (Manca [24], Graham-Rowe [25], Bunger [29], Moullin [30], Newlands [31], Templeton [32]).

#### Design

Nine of the 12 completed studies and protocols had observational designs; one protocol and one completed study were randomized controlled trials that incorporated a process evaluation (Prior [23], Elouafkaoui [33]). One protocol was a systematic review (Graham-Rowe [25]).

#### Methods

Two studies’ (Murphy [27], English [28]) sole objective was intervention design, using environmental scans, a priori knowledge about context, and a theory-informed approach. One study was quantitative (Bunger [29]), and two completed studies and four protocols were mixed-method (Gould [22], Prior [23], Manca [24], Sales [26], Templeton [32], Elouafkaoui [33]) including interviews, questionnaires, observation, comparative effectiveness, framework analysis, and network analysis. (Note that Gould et al. used CFIR + TDF in only one of their three sub-studies in which only qualitative (interview and observation) methods were used [22].) Two studies were qualitative (Moullin [30], Newlands [31]), and one protocol was a systematic literature review (Graham-Rowe [25]).

#### Data collection

Two completed studies (Murphy [27], English [28]) designed interventions; their data sources included results from authors’ previous and ongoing research (Murphy [27], English [28]), the published literature (Murphy [27], English [28]), authors’ cumulative tacit knowledge and experience (Murphy [27], English [28]), and informal discussions with stakeholders (English [28]); notably, both intervention development studies’ authors described CFIR + TDF as data sources. A third completed study (Bunger [29]) applied neither the CFIR nor the TDF during data collection. One protocol (Manca [24]) and one completed study (Moullin [30]) planned to collect qualitative interview data but did not state how CFIR + TDF would be used during data collection. Three protocols and two completed studies (Gould [22], Prior [23], Sales [26], Newlands [31], Elouafkaoui [33]) developed or planned to develop (Sales [26]) interview topic guides using CFIR + TDF, and one completed study developed a provider questionnaire using CFIR + TDF (Templeton [32]); however, none fully specified which constructs or domains would be used or how constructs or domains would be selected. One protocol, the systematic review (Graham-Rowe [25]) indicated that the authors would include some terms related to TDF domains in their search strategy, but they did not specify TDF domains.

#### Data analysis

Two completed intervention studies (Murphy [27], English [28]) indicated that they used CFIR + TDF not explicitly for data analysis but rather to promote their intervention’s implementation. English et al. indicated that they primarily used CFIR’s intervention domain, considering its constructs in the design and packaging of their intervention; they identified the TDF’s skills, social/professional role identity, reinforcement, goal, and social influence constructs as particularly relevant for informing the design of their intervention [28]. Murphy et al. used CFIR + TDF to “consciously give priority to the process of implementation within the design of the intervention” [27]. Specifically, the CFIR offered them a “meta-view” of constructs to consider that were relevant to the implementation of their intervention. How Murphy et al. used the TDF in data analysis was less clear, although the authors indicated that they considered the TDF during their assessment of pharmacists’ capability, opportunity, motivation, and behavior (COM-B) related to the intervention [27].

Two protocols (Gould [22], Prior [23]) and one completed study (Elouafkaoui [33]) were explicit in stating that CFIR + TDF would be (Gould [22])/was (Prior [23], Elouafkaoui [33]) used to code interview data, but none of these identified specific constructs or domains from each framework that would be used for analysis. Two studies used the TDF (not CFIR) to code interview data (Newlands [31], Templeton [32]). Moullin et al. used an adapted version of the CFIR, augmented with elements of the TDF, in a secondary analysis of interview data [30]. Graham-Rowe et al. planned to use all domains of CFIR + TDF to code data abstracted from their systematic review of existing literature [25]. Bunger et al. stated that CFIR + TDF provided theoretical justification for components of the learning collaborative intervention that they described, identifying specific constructs and domains from each framework that related to intervention components [29]. As with their explanation of how CFIR + TDF would inform data collection, two protocols were not explicit in stating whether and how CFIR + TDF would be used during data analysis (Manca [24], Sales [26]).

#### Unit of analysis

One protocol’s and one completed study’s units of analysis were the individual (Manca [24], Newlands [31]), and two protocols’ and one completed study’s units of analysis were the organization (Gould [22], Prior [23], Elouafkaoui [33]). Three completed studies and two protocols analyzed data at both individual and organizational levels (Graham-Rowe [25], Sales [26], Bunger [29], Moullin [30], Templeton [32]). As noted above, two completed studies’ sole objective was intervention design; although the intervention was targeted at healthcare professionals, environmental scan efforts were conducted in both studies, focusing on the societal, organizational, and individual levels (Murphy [27], English [28]). Five studies (Gould [22], Prior [23], Murphy [27], Templeton [32], Elouafkaoui [33]) and two protocols (Graham-Rowe [25], Sales [26]) referred to CFIR + TDF’s ability to tap the multiple levels at which implementation determinants lie, generally suggesting that the TDF tapped individual levels and the CFIR tapped collective levels.

Outcomes assessed. In contrast to other study characteristics, we only report outcomes assessed for study objectives that used CFIR + TDF. One completed study assessed healthcare professionals’ behavior (i.e., engagement in professional networks (Bunger [29]); two protocols assessed specific beliefs relating to a behavior (e.g., response to feedback from blood transfusion audit and feedback; Gould [22], Prior [23]); two protocols assessed intervention reach, effectiveness, adoption, implementation, and maintenance (Manca [24], Sales [26]); and three protocols and three studies assessed determinants of implementation (e.g., acceptability of the intervention; Gould [22], Prior [23], Moullin [30], Newlands [31], Templeton [32]). One study assessed the role and importance of CFIR + TDF domains (Graham-Rowe [25]). Two completed studies did not assess outcomes, as their sole objective was intervention design (Murphy [27], English [28]).

### Stated rationale for using CFIR + TDF

Table 2 excerpts studies’ descriptions of their use of CFIR + TDF and, in most cases, their stated rationale for using CFIR + TDF; four studies did not explicitly state their rationale for using CFIR + TDF (English [28], Bunger [29], Newlands [31], Elouafkaoui [33]).

**Table 2:** Studies’ rationale for using CFIR + TDF

Study	Rationale for using CFIR + TDF^a^	Purpose	Conceptual level	Degree of theoretical heritage	Operationalizability
Bunger et al. [29]	“We highlight the theoretical justification for the different components of the [learning collaboratives]…the [CFIR]…and the [TDF]. These frameworks highlight many important constructs that may need to be addressed in implementation efforts.” (p. 85)	No stated rationale			
Elouafkaoui et al. [33]	“The [CFIR] and the [TDF] for health psychology were used as coding frameworks.” (p. 9)	No stated rationale			
English [28]	“[The CFIR and TDF] were used to explore how and why potential intervention activities might be valuable in influencing hospital practice change. This helped to identify [intervention activities] felt to address core problems and that might both fit the context and support the overall effectiveness of a package of activities.” (p. 6)	No stated rationale			
Gould et al. [22]	“[U]se of the TDF to identify potential barriers to change individuals’ behaviour, may not be the only approaches to improving transfusion practice or optimising A&F in the hospital context. Behaviour change within a healthcare setting is a complex process, and due to the multi-level nature of healthcare organisations, elements of change in response to feedback may be outside the control of any individual healthcare professional… The [CFIR] provides a framework for identifying what works where and why across different organisational levels within multiple settings.” (p. 3)		x		
Graham-Rowe et al. [25]	“The [TDF]…[includes] theoretical domain[s] represent[ing] a range of related constructs that may mediate behaviour change at the level of the individual, team or healthcare organisation…. However, it is possible that barriers and enablers could operate at multiple levels in the healthcare system…The [CFIR]…offers a framework of theory-based constructs as a practical guide for systematically assessing potential barriers and facilitators to successful implementation across different organizational levels.” (p. 2)		X		
Manca et al. [24]	“The TDF is a comprehensive framework that includes all of the important constructs of implementation. Since it is inclusive and addresses a large number of domains (14) and constructs (84), it may not be the best tool to identify and prioritize the key elements of the implementation. However, an awareness of the constructs in the TDF will help ensure that no important construct is missed during the qualitative evaluation…The CFIR framework is a pragmatic synthesis of several frameworks and models and will inform the implementation process by identifying key elements in the program implementation in a systematic way.” (p. 7)	X			
Moullin et al. [30]	“Factors [influencing professional service implementation in community pharmacy] were assessed at each stage of implementation using the [CFIR]. CFIR was augmented with factors not included, or implied within broad constructs of the framework, in order to make them more explicit. Additional factors included behavioural influences from Theoretical Domains Framework.” (p. 4)		X		
Murphy et al. [27]	“[T]he CFIR provided a foundation for a meta-view of understanding important variables to consider with the implementation of a complex intervention designed for changing behaviour vis-a-vis community pharmacists in mental health care… We then followed a step-wise approach…to intervention design and development using the body of work by Michie and colleagues [including the TDF] to organize and conceptualize strategies to change behaviours.” (p. 2)	X			
Newlands et al. [31]	“Section 1 of the interview related to participants’ experiences and responses to the *RAPiD trial audit and feedback intervention* and were based on the…[CFIR]. Section 2 of the interview used a topic guide based on the TDF to explore the factors influencing *GDPs’ management* of patients with bacterial infections.” (p. 2)	No stated rationale			
Prior et al. [23]	“[U]sing the [CFIR] to explore the acceptability of the *interventions* and the [TDF] to identify barriers and enablers to evidence-based antibiotic prescribing *behaviour by GDPs*… [The TDF] allows for consideration of a comprehensive range of potential influences on health professional behavior… The CFIR consists of common constructs from published implementation theories and offers an over-arching typology to promote implementation theory development and verification to understand the mechanism about what works, where, and why across various contexts.” (p. 1; p. 7)	X	X		
Sales et al. [26]	“Our primary rationale for using both frameworks is that one (TDF) specializes in individual-level behavior change, while the other (CFIR) focuses more on the organizational level, above the individual.” (p. 3)		X		
Templeton et al. [32]	“The [TDF] was used to identify and describe patient-, organization-, and system-level barriers and facilitators to care…[Practice characteristics] were selected using the [CFIR] as a complement to the TDF to increase specificity of organizational assessment.” (p. 1)		X		

*CFIR* Consolidated Framework for Implementation Research [5], *TDF* Theoretical Domains Framework [6], *CFIR + TDF* use of the CFIR and TDF

^a^Emphasis added

Of the eight studies that explicitly stated their rationale for using CFIR + TDF, three indicated that the CFIR and the TDF addressed different *purposes* (Prior [23], Manca [24], Murphy [27]). In each of the three studies, the authors described one framework as offering an overarching perspective on implementation determinants and the other as more conducive to translating findings into practical approaches to implementation. Interestingly, two of these studies (Prior [23] and Murphy [27]) described the CFIR as offering an overarching perspective on implementation determinants and the TDF as offering specific determinants of healthcare providers’ behavior, whereas the third study (Manca [24]) offered the opposite rationale, citing the TDF as including “all of the important constructs of implementation” and the CFIR as a framework that would assist in “identifying key elements in the program implementation in a systematic way.”

Six of the included studies that explicitly stated their rationale for using CFIR + TDF indicated that the CFIR and the TDF addressed different *conceptual levels* (Gould [22], Prior [23], Graham-Rowe [25], Sales [26], Moullin [30], Templeton [32]). In each of the studies that cited multiple conceptual levels as their rationale for using CFIR + TDF, authors suggested that the CFIR addressed the collective level and the TDF addressed the individual level. For example, Sales et al. wrote “Our primary rationale for using both frameworks is that one (TDF) specializes in individual-level behavior change, while the other (CFIR) focuses more on the organizational level, above the individual” [26].

None of the studies’ rationales for using CFIR + TDF related to accounting for multiple *theoretical perspectives* or adequately *operationalizing* key constructs.

## Discussion

The objective of this study was to elucidate scholars’ rationale for using CFIR + TDF by describing (1) studies that have used CFIR + TDF; (2) how they used CFIR + TDF (e.g., framing, data collection, analysis); and (3) their stated rationale for using CFIR + TDF. CFIR + TDF was applied to studies in several countries, of a range of healthcare interventions and at multiple intervention phases. In particular, several of the included studies used CFIR + TDF to describe context by assessing characteristics of individuals and of the organization within which they were embedded. They then used this information to design tailored strategies for implementing an evidence-based practice. This reflects the generally held belief that tailoring strategies to context will lead to more effective implementation [34–36]. CFIR + TDF was also applied to studies with a variety of designs and methods, with multiple units of analysis and outcomes assessed.

Eight of the 12 studies included in our analysis explicitly stated a rationale for using CFIR + TDF. Three studies (Prior [23], Manca [24], Murphy [27]) suggested that the CFIR and the TDF addressed multiple study *purposes*. Authors of both the CFIR and the TDF describe the respective frameworks as a set of determinants to facilitate understanding implementation. In addition, Damschroder et al. suggested that the CFIR was intended to promote theory development and facilitate synthesis of research findings across studies and contexts [5], and Michie et al. suggested that the TDF may be used to promote the development of interventions to enhance implementation [6].

Differences in the purposes that authors of the CFIR and the TDF propose help to justify the rationale that these frameworks address multiple study *purposes*. Murphy et al. suggested that as a comprehensive framework, the CFIR provided a “meta-view” of implementation, whereas the TDF helped to conceptualize behavior change interventions [27]. Prior et al. similarly conceived of the CFIR as an “over-arching typology” for understanding implementation and the TDF as a lens for understanding provider behavior [23]. Manca et al. viewed the TDF as a comprehensive framework for understanding implementation but used the CFIR to inform the implementation process (a CFIR domain) [24].

Six studies (Gould [22], Prior [23], Graham-Rowe [25], Sales [26], Moullin [30], Templeton [32]) suggested that the TDF and the CFIR addressed different *conceptual levels* of implementation determinants. Damschroder et al. characterized the CFIR as a “comprehensive taxonomy of specific constructs related to the intervention, inner and outer setting, individuals, and implementation process” [5]. The TDF also includes constructs at multiple levels, including the individual and collective [6]. Given that the CFIR and the TDF both include determinants at both the individual and collective levels, the dichotomy that these six studies suggest may reflect potentially misleading conventional wisdom. To the extent that the CFIR and the TDF each sufficiently address constructs at the individual and collective levels, studies that use CFIR + TDF to address multiple conceptual levels may introduce unnecessary complexity and redundancy. On the other hand, it is possible that the CFIR and the TDF, when combined, help to more fully define the multi-level nature of behavior change in healthcare organizations than either of these frameworks alone. Formal efforts to map the CFIR and the TDF onto one another may help to explicate the extent to which using CFIR + TDF in fact helps to address multiple conceptual levels, or if using CFIR + TDF introduces unnecessary complexity and redundancy.

Four of the studies included in our analyses did not explicitly state a rationale for using CFIR + TDF; however, in some cases, how these studies used CFIR + TDF offered insight into their authors’ rationales for doing so. English et al. indicated that they primarily used the CFIR’s intervention domain and the TDF’s skills, social/professional role and identity, reinforcement, goals, and social influences constructs. The selection of these domains and constructs may suggest that English et al., like six other included studies, viewed the CFIR as a representative of the collective level and the TDF as a representative of the individual level: The CFIR’s intervention domain lies at the collective level, whereas the TDF constructs that English et al. included, such social/professional role and identity lie at the individual level. Similarly, Newlands et al. used the CFIR to collect data regarding the intervention (collective level) and the TDF to collect data regarding provider behavior (individual level), and in Elouafkaoui et al.’s [33] description of their unit of analysis, they suggested that TDF tapped individual levels and the CFIR tapped collective levels.

Our finding that several included studies did not explicitly state a rationale for using CFIR + TDF is consistent with other studies that have found inadequate descriptions and justifications for included frameworks in empirical studies [11, 37–39]. This underscores the need for guidance on how to apply frameworks and report their use, perhaps signaling the need for specific reporting standards such as those developed for implementation strategies [38, 40].

## Implications

In fulfilling our objective of elucidating scholars’ rationale for using CFIR + TDF, we aimed to inform the judicious use of CFIR + TDF and combinations of other frameworks in future implementation studies. Doing so may help to limit the proliferation of frameworks and the related fragmentation of knowledge by ensuring that existing frameworks continue to be used, evaluated, and refined and by avoiding segmentation of the field through use of a single preferred framework over another [1, 17, 18]; curtail “pseudoinnovation,” wherein perceived advances in framework development are more aptly characterized as reinvention rather than true innovation [18]; yield more practically relevant study results; and move implementation science toward greater interdisciplinarity.

We found that eight of the 12 included studies indicated that they used CFIR + TDF to address multiple study *purposes* and *conceptual levels*. Specifically, authors of six included studies suggested that either the CFIR or the TDF offered an overarching perspective on implementation determinants, whereas the other was described as more conducive to translating findings into practical approaches to implementation. And authors of six included studies suggested that the CFIR and the TDF addressed distinct conceptual levels; generally speaking, these studies argued that the TDF addressed multiple conceptual levels but did not sufficiently elaborate on determinants of implementation at the collective level. To avoid concerns regarding unnecessary complexity or redundancy, scholars who use CFIR + TDF and combinations of other frameworks as useful for addressing multiple study needs should explicitly specify the benefits of each included framework; doing so will help to justify the use of multiple frameworks, and it will contribute to our understanding of each framework, its scope, and its limitations.

Our finding that some included studies did not explicitly state a rationale for using CFIR + TDF suggests that, at a minimum, future studies that apply multiple frameworks should clearly describe the ways in the frameworks, when combined, contribute to their study; the ways in which each framework alone is limited in contributing to their study; how the frameworks’ contributions converge and/or diverge; and recommendations for the refinement of one or more framework. Doing so will strengthen the studies and contribute generalizable knowledge regarding the frameworks.

## Limitations

A few limitations of this systematic review should be noted. Although we searched many databases with wide coverage using clear, specific, and appropriate terms, we included only peer-reviewed published research in English. It is possible that the search did not yield all published studies that used CFIR + TDF. For example, additional applications may be found in “grey literature” or in different languages. In addition, our ability to understand scholars’ rationale for CFIR + TDF was limited to the extent that they did not explicitly state their rationale; however, how scholars used CFIR + TDF offered insight into their rationale. A future study that interviews scholars regarding their rationale for using CFIR + TDF may provide additional insight.

## Conclusions

The findings from this review indicate that it is not uncommon for implementation researchers to use CFIR + TDF to inform their work. As future studies use CFIR + TDF and combine other frameworks, there is a need to answer fundamental questions about whether and how the use of multiple frameworks yields substantial benefits beyond that of a single framework applied to its full potential. This review also echoes findings from across the field of implementation science that the explicit use of theories and frameworks needs improvement [39]. Given the lack of guidance in the field, we call for the development of practical tools to guide the application of theories and frameworks as well as corresponding reporting guidelines. These efforts will help to clarify the application and contribution of theories and frameworks in implementation research and will facilitate richer theoretical understanding of implementation determinants, processes, and outcomes.

## Additional file

Additional file 1: PRISMA checklist. (DOC 59 kb)

## Abbreviations

CFIR: Consolidated Framework for Implementation Research
CFIR + TDF: Consolidated Framework for Implementation Research and Theoretical Domains Framework
PROSPERO: Prospective Register of Systematic Reviews
TDF: Theoretical Domains Framework
